# An Impartial Semi-Supervised Learning Strategy for Imbalanced Classification on VHR Images

**DOI:** 10.3390/s20226699

**Published:** 2020-11-23

**Authors:** Fei Sun, Fang Fang, Run Wang, Bo Wan, Qinghua Guo, Hong Li, Xincai Wu

**Affiliations:** 1 School of Geography and Information Engineering, China University of Geosciences, Wuhan 430078, China; sunfei@hgnu.edu.cn (F.S.); fangfang@cug.edu.cn (F.F.); wanbo@cug.edu.cn (B.W.); lihong@cug.edu.cn (H.L.); wuxincai@cug.edu.cn (X.W.); 2 Academy of Computer, Huanggang Normal University, No. 146 Xinggang 2nd Road, Huanggang 438000, China; 3 National Engineering Research Center for Geographic Information System, China University of Geosciences, Wuhan 430078, China; 4 Key Laboratory of Geological Survey and Evaluation of Ministry of Education, China University of Geosciences, Wuhan 430078, China; 5 State Key Laboratory of Vegetation and Environmental Change, Institute of Botany, Chinese Academy of Sciences, Beijing 100093, China; qguo@ibcas.ac.cn

**Keywords:** image classification, class imbalance, impartial semi-supervised learning strategy (ISS), extreme gradient boosting (XGB), very-high-resolution (VHR)

## Abstract

Imbalanced learning is a common problem in remote sensing imagery-based land-use and land-cover classifications. Imbalanced learning can lead to a reduction in classification accuracy and even the omission of the minority class. In this paper, an impartial semi-supervised learning strategy based on extreme gradient boosting (ISS-XGB) is proposed to classify very high resolution (VHR) images with imbalanced data. ISS-XGB solves multi-class classification by using several semi-supervised classifiers. It first employs multi-group unlabeled data to eliminate the imbalance of training samples and then utilizes gradient boosting-based regression to simulate the target classes with positive and unlabeled samples. In this study, experiments were conducted on eight study areas with different imbalanced situations. The results showed that ISS-XGB provided a comparable but more stable performance than most commonly used classification approaches (i.e., random forest (RF), XGB, multilayer perceptron (MLP), and support vector machine (SVM)), positive and unlabeled learning (PU-Learning) methods (PU-BP and PU-SVM), and typical synthetic sample-based imbalanced learning methods. Especially under extremely imbalanced situations, ISS-XGB can provide high accuracy for the minority class without losing overall performance (the average overall accuracy achieves 85.92%). The proposed strategy has great potential in solving the imbalanced classification problems in remote sensing.

## 1. Introduction

Classification with an imbalanced sample set is very common in real-world scenarios [1]. Many thematic classification maps can only be trained, calibrated, and validated with imbalanced samples due to the high costs of obtaining labels and the lack of ancillary information for sampling [2]. However, these imbalanced samples often pose difficulties for learning algorithms. Because classifiers are biased towards the majority class [3], the minority class, which may be a category of concern for researchers, is omitted under this situation [4]. Inaccurate results introduce potential risks for decision-making and industry applications. Moreover, imbalanced learning is an issue that is often ignored in land-use and land-cover classification.

Studies on class imbalance problems can be summarized based on three relevant tasks: processing training data, improving the classification algorithm, and constructing a learning strategy [5]. From a data perspective, resampling approaches that include resampling in data space and feature space have been widely adopted and researched. In the data space, the under-sampling method [3,6,7,8], the over-sampling method [9,10], and a combination of the two [11,12] are often used to balance the sample sizes of different classes. Tomek link [13], NearMiss [14], and other variant methods are representative of under-sampling approaches. For skills, synthetic techniques [15,16,17] that join different weights when sampling [18] are commonly used during the resampling process. Among the various over-resampling techniques used in data space, the SMOTE (synthetic minority over-sampling technique) [16] method and its variants, such as Borderline-SMOTE [19] and Adaptive Synthetic Sampling (ADASYN) [20], have been widely applied in academia and industry [21]. Meanwhile, feature selection—including filters, wrappers, and embedded methods—can be regarded as a resampling technique implemented in the feature space, which is also a solution for imbalanced learning issues [22,23]. However, choosing an appropriate method at the data level is complicated [24,25]. Waldner [26] proposed an algorithm called F-race to identify an optimal or sub-optimal resampling-based balancing method via interactive selection.

At the algorithm level, many methods and variants aimed at imbalanced learning have been put forward. Cost-sensitive learning [27,28,29] and ensemble learning [11,30,31] are the most widely used approaches. The former requires a cost matrix to adjust the error distribution of the classifier on different classes during the training process. The latter is a strong classification system that combs several base classifiers to generate predictions using specific generalization rules [25]. Moreover, various models and modifications make the imbalanced training process more diversified, such as optimization in kernel and activation functions [32], the mixed objective function method [33,34], fuzzy theory predictions [35], and clustering [36]. The random forest (RF) [37] and support vector machine (SVM) [38] algorithms have been used and optimized to predict tree classes in remote sensing data. The novel extreme gradient boosting (XGB) technology has also been used to assess imbalanced land-cover classification [39]. In addition to the single perspectives mentioned above, other solutions have combined multiple perspectives—for example, Li et al. [40] employed a cost-effective network extension scheme for the convolutional neural network (CNN) to classify vehicle objects in very-high-resolution (VHR) images. Krawczyk [41] and Hassan [42] adopted ensemble learning and under-sampling for breast cancer malignancy grading and automobile insurance fraud detection, respectively. In general, data-level approaches are more frequently used in practice [25]. These approaches provide a more direct and convenient way to handle imbalanced data without modifying the learning algorithm. 

From a strategy perspective, imbalanced problems are transformed to a relatively balanced problem by redefining of the problem space or learning strategy. This kind of method mainly uses the idea of decomposition. For example, ensemble learning combined with a one-on-one learning strategy [43], pairwise learning with fuzzy rules [44], or learning with a hierarchical data structure [45]. One-class classification can deal with imbalanced learning problems by reducing the type and number of the sample requirements [46].

However, although numerous solutions exist, it remains difficult to directly find the most appropriate solution for imbalance issues. When adopting the cost-sensitive learning method, the appropriate cost matrix is not easy to determine, even for experts [27]. The resampling method is direct and simple, but the associated resampling approach is not fixed [25]. Each approach has its own advantages and disadvantages. Under-sampling may lose potentially useful information, oversampling may result in overfitting with a large number of duplicate samples, and a synthesized sample cannot correspond to actual instances in remote sensing images. Some complex algorithms, such as CNN, require large computational resources [40,47]. A good solution is to establish an unbiased strategy that eliminates skewed distribution during training without increasing the sample’s labeling cost. Data and class decomposition can break up the data distribution of problems into multiple minority or majority classes.

Semi-supervised learning methods, which explore the hidden distribution information from unlabeled data for learning, may be effective methods for solving this difficulty [48]. Positive and unlabeled learning (PU learning), proposed by Elkan and Noto [49], is one of the best semi-supervised methods for remote sensing data [50]. This semi-supervised method handles the classes one by one (OBO), which is different from the traditional all-in-one (AIO) framework (i.e., where the samples of all classes are combined to train the classifier at the same time) [50]. The unlabeled data in PU learning can provide the distribution information of the covariates for model training [51] and act as a regularizer to prevent overfitting [48]. However, in the remote sensing field, this method has been reformed for class-incomplete issues [50,52] or binary classification applications [51,53,54] but not for sample imbalance problems.

In this paper, we proposed an impartial semi-supervised learning strategy based on PU learning for sample imbalanced issues in remote sensing. This strategy utilizes unlabeled data to convert a positive–positive (traditional all-in-one methods with all positive samples) classification scheme into a positive–unlabeled scheme and compensated for the learning deficiency caused by the extreme rarity of samples through multiple positive–unlabeled training. In this strategy, a simulator is needed for the positive-unlabeled training, such as the back-propagation neural network (BP) [50,52,53] or support vector machine (SVM) [51,55]. However, it is known that BP and SVM are sensitive to extremely rare samples, which may lead to simulation bias [1,3,25]. Extreme gradient boosting (XGB) has proven to be more predictive than non-boosting methods and uses iterative gradient boosters for regression simulation with limited samples [56,57]. Thus, the proposed impartial semi-supervised learning strategy employs XGB (an impartial semi-supervised learning strategy based on extreme gradient boosting, ISS-XGB) as a simulator to improve the simulations and predictions for the positive class. 

The rest of this paper is organized as follows. Section 2 describes the principle of ISS-XGB for imbalanced learning. Section 3 outlines experiments conducted on two VHR remote sensing data resources across eight study areas with different complexities. Section 4 demonstrates the performance of ISS-XGB with imbalanced samples. Section 5 further evaluates the effectiveness of the proposed strategy via two contrastive analyses: (1) a comparison with previous semi-supervised models in remote sensing and (2) a comparison with synthetic resampling technique-based methods. The influence of unlabeled data on ISS-XGB is also discussed in this section.

## 2. The Principle of ISS-XGB: Impartial Semi-Supervised Learning Strategy for Imbalanced Learning

ISS-XGB is an impartial semi-supervised learning strategy based on extreme gradient boosting. It is built on the PU learning framework, and models are trained with positive samples and are randomly selected as unlabeled data through the OBO strategy.

Suppose that x represents the covariates associated with an instance and y represents the property of the instance, where y=1 denotes positive data, and y=0 denotes negative data. The target of classification can be defined as a function of the probability that a pixel is positive based on its characteristics (or covariates), denoted as f(x)=p(y=1|x). 

**Figure 1:**
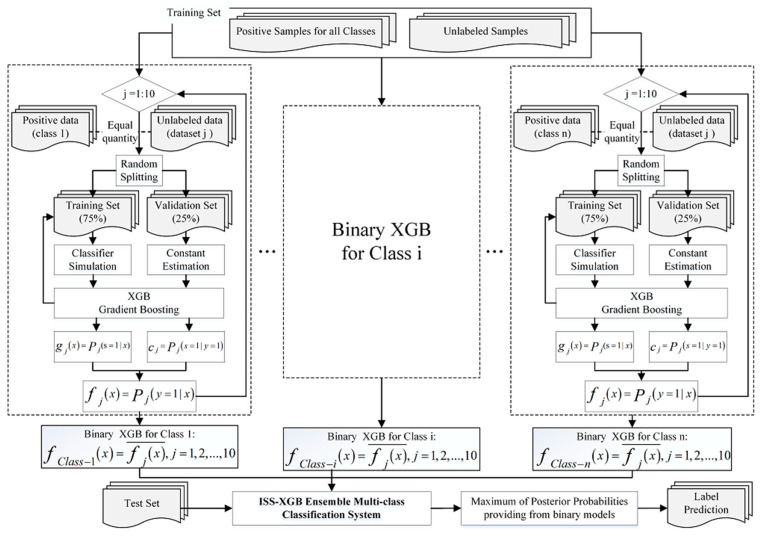
Workflow of the impartial semi-supervised learning strategy based on extreme gradient boosting (ISS-XGB) ensemble algorithm.

However, in ISS-XGB, the input datasets for each training procedure are only the positive samples of a certain class and unlabeled data (s=1 represents labeled, and s=0 represents unlabeled). Thus, for certain class i, the learner is a positive–unlabeled simulator  g(x)=p(s=1|x), which simulates the probability that a pixel is labeled. For n-class classification problems, n positive–unlabeled models are generated (Figure 1). The imbalanced learning problem is converted to multiple positive–unlabeled problems. Such decomposition strategies can help to avoid inputting positive samples of minority classes and majority classes into the simulator at the same time. 

Since only positive data can be labelled, the probability of a negative datum to be labeled is zero:

(1) p(s=1|x,y=−1)=0

The labeled samples are chosen randomly from all positive pixels; thus, the probability that a positive pixel is labeled is a constant “c” regardless of *x* is as follows:

(2) p(s=1|x,y=1)=p(s=1|y=1)=c

With Equations (1) and (2), we have

(3) $$\begin{array}{ll} g(x) & =p(s=1|x)\\ & =p(y=1\wedge s=1|x)\\ & =p(y=1|x)p(s=1|y=1,x)\\ & =p(y=1|x)p(s=1|y=1) \end{array}$$

Therefore, there is a clear relationship between f(x) and g(x):

(4) f(x)=g(x)/c.

Li [52] provided an approach to obtain c by using an average of the predicted probabilities of multiple positive pixels in a validation set. More detailed proofs can be found in [49].

The PU learning framework takes advantage of randomly selected unlabeled data by employing auxiliary information of the positive sample distribution to simulate the target. Research based on this framework often tends to use a large amount of unlabeled data (5000 in [51,52]) to provide sufficient information. However, an inconsistent number of positive and unlabeled samples can still lead to imbalanced learning issues, especially when the positive samples are extremely rare. Thus, in ISS-XGB, equal quantities of positive samples and unlabeled datasets are used for training a balanced one-class classifier (Figure 1). Moreover, to obtain enough information on the whole distribution and ensure the stability of the simulation, an ensemble of multiple unlabeled datasets (10 times in this paper) is created for each class. In this way, the target f(x) can be identified as $f_{j}\left(x\right)$, where j=10. Ten simulators ($g_{j}\left(x\right)$) and c_j_ are integrated into a sub simulation system $f_{class-i}\left(x\right)$. The final posterior probability is calculated by the mean of the probability output, i.e., $f_{class-i}\left(x\right)=\bar{f_{j}\left(x\right)}$, representing the probability of an instance belonging to class *i*. The final label is assigned based on the maximum posterior probability of each class (Figure 1). 

The simulator g(x) is trained by a binary classifier, XGB. This is a scalable regularized gradient boosting technology that provides predictive performance. As an ensemble tree model, XGB uses iterative gradient boosters to construct a strong classification model. XGB has shown predictive abilities for binary classification problems [57,58] and land-cover classification problems [39,47,59,60]. The gradient correction of XGB helps classifier learning and constant estimation from imperfect representations of limited samples. The gradient correction of XGB is helpful for classifier learning and constant estimations from the imperfect representation of limited samples. 

In summary, ISS-XGB has two main optimizations aimed at imbalance issues. First, equal amounts of the training data (positive and unlabeled) prevent the inner imbalance of the sample sets. Meanwhile, multiple unlabeled datasets are used to offer substantial information on the distribution. Second, XGB is employed as the simulator to improve the certainty of simulation via iterative gradient boosters. It is worth noting that the unlabeled data are randomly selected from the images. ISS-XGB can handle the imbalanced land-cover classification problem without extra labeling costs. 

For n-class classification problems, the core idea of ISS-XGB is to use positive-unlabeled framework to transform the tasks into n-binary learning components. Each positive-unlabeled sub-component consists of 10 PU training routines. To compensates for learning deficiency caused by sample imbalance, every routine is based on the same number of positive samples and unlabeled data. To establish the relationship (estimate c) between f(x) and g(x), the training data in every routine is split, 75% for training, and 25% for validation. Random splitting and multiple routines with different unlabeled data can also help control overfitting. According to Equation (4), the conditional probability of one routine is estimated by XGB. Then the average of 10 routines provides the estimations of current class i. All n $f_{class-i}\left(x\right)$ constitute the ISS-XGB ensemble model. Moreover, ISS-XGB adopts maximum probabilities rule for label prediction (Figure 1). 

## 3. Data and Experiment

### 3.1. Study Areas and Data

**Figure 2:**
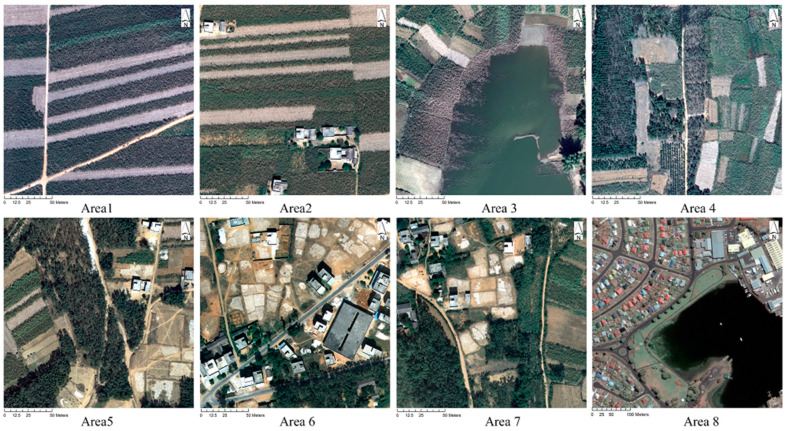
Study areas. Areas 1 to 7 are aerial images with 3 bands (R: 610-660 nm; G: 535–585 nm; and B: 430–490 nm), Area 8 comprises GeoEye-1 images with 4 bands (R: 675 nm; G: 545 nm; B: 480 nm and NIR: 850 nm). Each area is limited to 1000 × 1000 pixels. Therefore, the numbers of positive samples are limited to within 1.1% of the whole population (0.3%, 0.5%, 0.6%, 0.5%, 0.6%, 0.6%, 0.7%, and 1.1%, respectively).

Eight study areas with different complexities were selected. Areas 1–7 (aerial images acquired by ADS40 in 2014 with a 0.2 m spatial resolution) are located in Beihai, Guangxi, a normal suburban area in China. Area 8 (GeoEye-1 data from the environment for visualizing images (ENVI) tutorial data, an open-access data source acquired in 2009 with a 0.5 m spatial resolution) is located in northwest Hobart (Tasmania, Australia). The landscape distributions of all 8 areas exhibit different class imbalances without uncertain shadows caused by high buildings. (Figure 2)

All datasets use VHR data, which is beneficial for random sampling and evaluating the performance of different methods [50]. VHR allows manual image interpretation to be carried out for labeling land-cover types with higher confidence than that achieved with coarser resolution images. For accuracy verification, manual interpretation was used to obtain the pixel labels. Based on China’s National Standard “Current Land-use Classification, GB/T 21010-2017” and ancillary data (the production of Second National Land Survey for Beihai), areas 1–7 cover several typical land classes: house, road, tree grass, soil, water, farmland, and others. Meanwhile, the farmland class was further divided into farmland with and without crops by manual interpretation, as to be consistent with the surface cover (Table 1). Referring to the same standards and labeling procedures above, area 8 was ultimately finally divided into 11 classes (Table 1), including building-1 with red roofs, building-2 with grey roofs, and building-3 with light green roofs in pseudo mode (R/G/B:4/3/2). Additionally, high-light objects, such as ground vehicles, surface vessels, etc., were classified as one type. In every study area, one land-cover class with a low proportion was specified as the minority class, and the rest were all majority classes (Table 1). Eight second-order texture metrics (mean, variance, homogeneity, contrast, dissimilarity, entropy, second moment, and correlation) [50,52] for each spectral band were also used for training. All features were extracted in ENVI 5.1 with a 3 × 3 pixel template along the horizontal direction.

To quantify the complexity and diversity of the study areas, the Shannon diversity index (SHDI), which considers both species richness and abundance, was used [61]. This index was calculated using the entropy equation with the proportions of classes existing in each area. The SHDI of the 8 study areas ranged from 0.83 to 2.22 (acquired by Fragstats 4.2 [62]), meaning that the diversity of the areas ranged from relatively simple to complex.

### 3.2. Experimental Set-up and Accuracy Assessment

To evaluate the performance of ISS-XGB on different sample imbalances, 50 sample sets with different class proportions were randomly selected from each study area. Each sample set consisted of 1000 × n (where n represents the number of classes) samples, whose class proportion was a:b (a represents the minority class, and b represents the majority class). To generate different imbalanced sample sets, a was increased from 1 to 50, while b was altered in the reverse manner (from 99 to 50) [63], forming distributions ranging from extremely skewed to completely balanced. Here, we assumed that due to the large distribution of the majority classes, it was easy to form abundant positive sample data for the classes. Simultaneously, to reduce the impact of non-single changes in the number of samples on the study of imbalanced classification patterns, the majority classes in the experiments used a sample set with the same amount. Reproducible trials were conducted with datasets of different class imbalances to ensure statistically reliable accuracy. The final reported results for each accuracy measure represents the average values of 100 trials. Additionally, test sets were randomly selected from whole images before the selection of training samples.

The performance of ISS-XGB with different imbalanced samples was also compared with that of approaches dealing with imbalanced learning issues in the remote sensing and machine learning fields, such as MLP [64], RF [37], and XGB [58,65]. It was also compared with SVM [38], which used a binary training mode (one-vs.-rest strategy). However, SVM and ISS-XGB are quite similar in the learning framework. The most significant difference is that SVM used negative samples, but ISS-XGB used unlabeled data. Moreover, typical semi-supervised learning methods in remote sensing, including PUL [52] (PU-BP in this paper) and PUL-SVM [55] (PU-SVM in this paper), were also compared with the proposed strategy to develop on insightful analysis of the optimizations in ISS-XGB. In addition, SMOTE [16], which uses relative positive neighbors to synthesize extra samples for the minority class, is a popular and direct approach to deal with imbalanced learning issues [25]. Thus, we also explored the performance of ISS-XGB and SMOTE-based methods.

To obtain complete insight into the performance of this multi-class imbalanced learning scenario, not only the overall performance but also the accuracy of the minority class was quantified. The overall accuracy (OA), Cohan’s Kappa coefficient (κ), and user and producer accuracy are the most frequently used measures in the remote sensing field [66]. However, Pontius (2011) indicated that κ only focused on the agreement and evaluated accuracy using randomness as a baseline, which was useless for explaining the error. Thus, quantity disagreement (QD) and allocation disagreement (AD) were recommended to assess disagreement performance [67]. Moreover, the recommended disagreement measures [67] could convert the observed sample matrix into an estimated unbiased population matrix to assess the performance of the population (this aspect is ignored in many applications). Thus, this paper used QD and AD to quantify the disagreement of the label prediction. The F_1_ score [25] represented the harmonic mean of user and producer accuracy, which reached its best value at 1 and worst at 0. Therefore, we used this indicator to evaluate the agreement of the minority class. To this end, we followed the recommendations and routine to calculate OA, QD, and AD (based on the population matrix [67]) to determine that overall performance and F_1_ score, QD’, and AD’ (based on the confusion matrix [67]) for the minority class. These measures were divided into two categories: agreement performances (OA, F_1_ score) and disagreement performances (QD, AD).

### 3.3. Parameter Optimization

The parameters of all methods were optimized in an empirical parameter space. For ISS-XGB and XGB, five main parameters were optimized, including the number of estimators (1~1000), learning rates (0.1~1), maximum tree depths (2~23), minimum leaf instances (1~29), and subsample rates (0.1~1). Similar to XGB, RF is composed of many simple decision trees as a strong model. To ensure the same complexity available in RF- and XGB-based methods, the number of simple trees was set to equal to the optimized number of estimators in XGB. The max_features parameter was set to the square root of the number of predictor variables [37,68]. For MLP and PU-BP, we used the same net structure [50], which mainly optimized the penalty parameter. This paper utilized the RBF kernel-based SVM, which is widely used in remote sensing classifications [38]. Cost parameters were mainly optimized on a validation set from balanced samples for every study area with a search scale of 1 to 200. For the multi-class classification scenario in this paper, the "one against the rest" mode was employed for SVM training. In addition, a data set with balanced positive and unlabeled samples is recommended for PU-SVM [55]. Therefore, the amount of unbalanced data in PU-SVM was set as equal to the positive samples of the current class, whereas 5000 unlabeled data were used in PU-BP, which is consistent with [50]. For SMOTE, the parameters of k (number of neighbors participating in synthesis) were set to 5, and the synthesis magnification was 100 (that is, equalization synthesis). All approaches were implemented using the scikit-learn package for Python 3.6 [69] (Windows 10, 3.40 GHz, and 8.0 Gb memory with an of Intel Xeon E3 CPU).

## 4. Results

Identification of the minority class is a difficult task in imbalanced classification. The proposed method (ISS-XGB) aimed to improve the accuracy of the minority class without losing the accuracy of the majority classes and the overall performance. Thus, we analyzed the performance of ISS-XGB to both the minority class and entirety class. In addition, we further investigated the effectiveness of ISS-XGB under different data complexities.

### 4.1. Performance on the Minority Class

**Figure 3:**
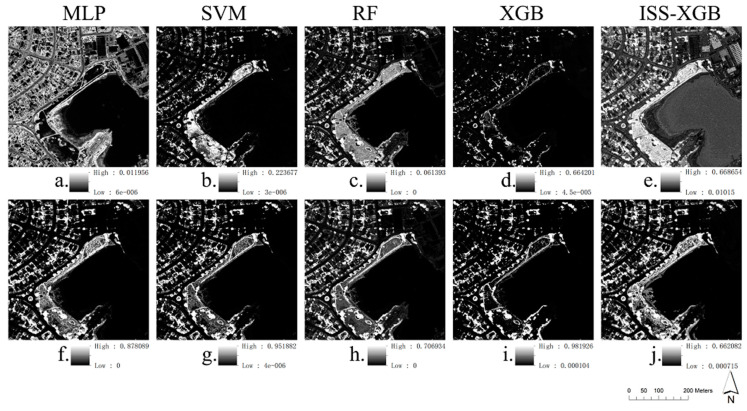
Predicted probability maps of the minority class (tree) of Area 8 with different imbalanced samples (minority:majority = 2:98; minority:majority = 50:50). Sub-figure (**a**–**e**) shows the predicted probability map by different methods with a sample ratio at 2:98 ((**a**): MLP; (**b**): SVM; (**c**): RF; (**d**): XGB; (**e**): ISS-XGB). Sub-figure (**f**–**j**) shows the predicted probability map by different methods with a sample ratio at 50:50 ((**f**): MLP; (**g**): SVM; (**h**): RF; (**i**): XGB; (**j**): ISS-XGB).

Area 8, which contained 11 land-cover species (Table 1) and possessed the highest complexity and diversity (SHDI = 2.22), was chosen as an example. We analyzed the performance of ISS-XGB, MLP, RF, XGB, and SVM on the minority class under two class proportions. The proportion rate (2:98) represented an extremely imbalanced situation, while the proportion rate (50:50) was the balanced situation. The probability maps of the minority class (i.e., tree) and the quantity measures are shown in Figure 3 and Table 2.

Under extremely imbalanced situations, the probability maps were significantly different (first row in Figure 3). The highest probabilities of MLP, SVM, and RF were only 0.012, 0.234, and 0.062, which may have led to a failure in identifying minority classes. Indeed, these methods omitted all tree pixels in Area 8 with a QD’ of 100% (Table 2). Both XGB and ISS-XGB provided higher probabilities (approximately 0.67 in Figure 3). However, XGB omitted most tree pixels with a QD’ of 99.64% and AD’ of 0% (Table 2). The F_1_ score of the minority class in XGB was only 0.01, while ISS-XGB was much higher, reaching 0.77 (Table 2). ISS-XGB successfully identified the minority class when the data distribution was extremely skewed.

Under a balanced situation, ISS-XGB can achieve the same performance as the commonly used methods. The probability maps of the minority class are visually similar (second row in Figure 3). The F_1_ scores of different methods are also very close and range from 0.82 to 0.86 (Table 2). However, the highest probability that ISS-XGB provides under different situations (Figure 3e,j) is more stable than other methods. 

This phenomenon is related to the role of unlabeled data in training. In different scenarios, unlabeled data has different importance to the description of class heterogeneity and the general information. The unlabeled data has different suppression effects in probability decision function in ISS-XGB. Therefore, in the situation with more balanced sample data, the probability estimates by ISS-XGB for the minority class do not increase as much as those of other methods (details in Section 5.1).

### 4.2. Overall Performance

**Figure 4:**
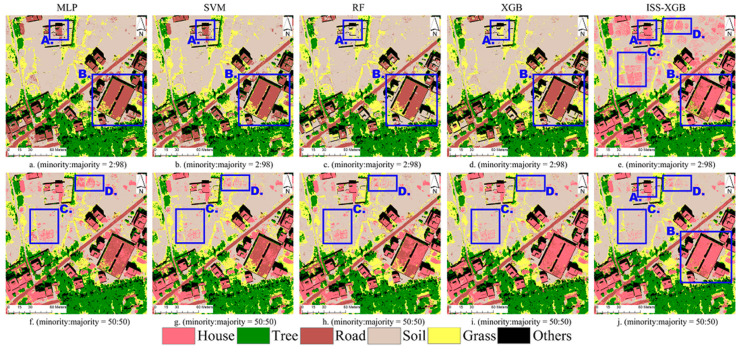
Classification maps of MLP, SVM, RF, XGB, and ISS-XGB with different imbalanced samples (minority:majority = 2:98 or 50:50). Sub-figure (**a**–**e**) shows the classification maps by different methods with a sample ratio at 2:98 ((**a**): MLP; (**b**): SVM; (**c**): RF; (**d**): XGB; (**e**): ISS-XGB). Sub-figure (**f**–**j**) shows the classification maps by different methods with a sample ratio at 50:50 ((**f**): MLP; (**g**): SVM; (**h**): RF; (**i**): XGB; (**j**): ISS-XGB).

**Figure 5:**
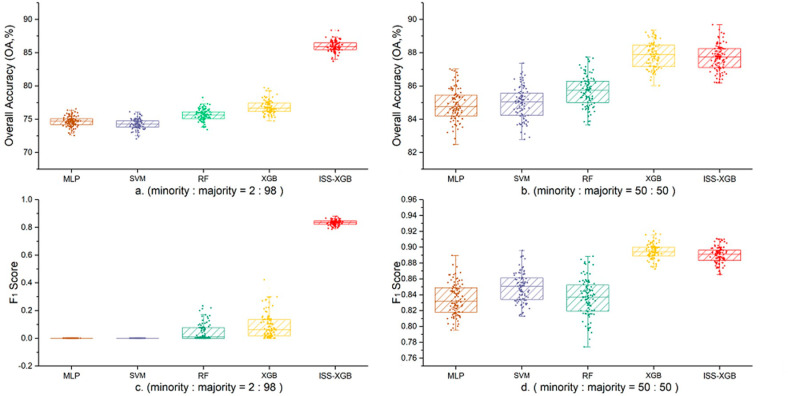
Box plots of OA and F_1_ scores for Area 6 with 100 trials using different imbalanced samples. Left Column: performance with samples at minority:majority = 2:98; Right Column: performance with samples at minority:majority = 50:50. Sub-figure (**a**,**b**) are the box plots on OA by five methods with different sample imbalance ((**a**). 2:98; (**b**). 50:50). Sub-figure (**c**,**d**) are the box plots on F_1_ scores by five methods with different sample imbalance ((**c**). 2:98; (**d**). 50:50).

As the contribution of the minority class to overall performance is limited in complex imbalanced datasets, a relatively simple area (i.e., area 6) was used as an example. This area, exhibiting an SHDI value of 1.43, contained six land-cover species: house, tree, soil, road, grass, and others. The minority class was the house. The visualization maps and accuracy results are shown in Figure 4 and Figure 5 and Table 3. A detailed confusion matrix of one trial can be found in Table 4. 

Under an extremely imbalanced situation, the difference in classification results was obvious (First row in Figure 4). ISS-XGB demonstrated better performance than the other approaches, providing an average OA of 85.92% (Figure 5a and Table 3). The mean F_1_ score of the minority class by ISS-XGB was 0.83, which was much higher than RF (0.04), XGB (0.09), MLP, and SVM (almost 0). The house pixels in parts A and B were successfully identified using ISS-XGB (Figure 4e), while the other four methods misclassified them as soil or roads (Figure 4a–d). 

Compared with the imbalanced situation, the performance with the balanced samples improved (Figure 4). Notably, the F_1_ scores of MLP, SVM, RF, and XGB increased by over 0.8 (Figure 5). The growth of the average OA and the F_1_ scores of ISS-XGB was low with less than 1.8% and 0.06, respectively (Figure 5). ISS-XGB offered comparable performance in both imbalanced and balanced cases. 

To analyze the statistical reliability of 100 trails, Table 3 shows the average and standard deviations (STD) of OA and F_1_ scores for the minority class under two different imbalance scenarios. Under an extremely imbalanced situation (minority:majority = 2:98), both in terms of OA and minority F_1_ scores, the ISS-XGB method showed more excellent accuracy and stability. Especially, the STD of the minority F_1_ scores via ISS-XGB was only 30% of that by RF, and 21% of the STD by XGB. It implied that ISS-XGB had better stability in this case than other methods. However, in imbalanced situations, ISS-XGB did not have obvious advantages in extremely imbalanced situations (Table 3).

The QD and AD of ISS-XGB with imbalanced samples were similar to those with balanced samples (Table 4). The slight increase with balanced samples was mainly due to a decrease of commission errors in the minority class (e.g., Parts C and D in Figure 4e,g). Balanced samples provided more helpful information for the minority class identification.

In general, ISS-XGB achieved a stable performance. Especially under extremely imbalanced situations, ISS-XGB provided high accuracy for the minority class without losing overall performance.

### 4.3. The Performance under Different Levels of Data Complexity

**Figure 6:**
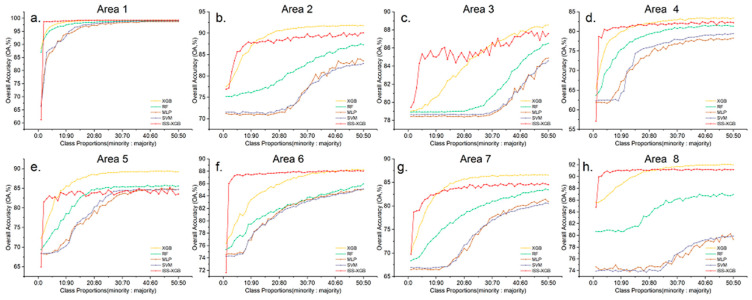
OA curves of all 8 areas for different imbalanced samples with ISS-XGB (red), XGB (yellow), RF (green), MLP (brown), and SVM (purple). Sub-figure (**a**–**h**) shows the OA curves by different areas ((**a**): Area 1; (**b**): Area 2; (**c**): Area 3; (**d**): Area 4; (**e**): Area 5; (**f**): Area 6; (**g**): Area 7; (**h**): Area 8).

**Figure 7:**
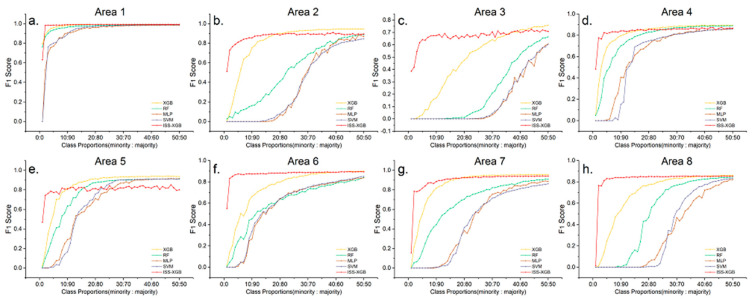
F_1_ score curves for the minority classes for all 8 areas across different imbalanced samples with ISS-XGB (red), XGB (yellow), RF (green), MLP (brown), and SVM (purple). Sub-figure (**a**–**h**) shows the F_1_ score curves by different areas ((**a**): Area 1; (**b**): Area 2; (**c**): Area 3; (**d**): Area 4; (**e**): Area 5; (**f**): Area 6; (**g**): Area 7; (**h**): Area 8).

To analyze the performance of ISS-XGB under different levels of data complexity, experiments were conducted in 8 areas across 50 different sample distributions (i.e., the class proportions were 1:99, 2:98, 3:97,…, 50:50). The curves of the OA and F_1_ scores are shown in Figure 6 and Figure 7.

Figure 6 and Figure 7 showed that when the samples were extremely imbalanced (i.e., the class proportion ranges from 2:98 to 10:90), the accuracy of ISS-XGB was higher than the other methods. For example, in area 2, the OA of ISS-XGB (Figure 6b) converged to 87.63% when the class proportion was 10:90, while other methods were close to or far below this value. MLP and SVM converged to approximately 82% when the sample distribution was balanced (50:50). RF generated better performance (87.31% at the class proportion of 50:50) than MLP and SVM but much worse performance than that of XGB and ISS-XGB. Although the maximum OA of XGB was slightly higher (90.19%), the requirements for the samples also increased. In either case, the OA of ISS-XGB always converged within a short interval (i.e., before the class proportion increases to 10:90). Moreover, this advantage of ISS-XGB was more obvious in terms of the F_1_ scores for the minority class (Figure 7); i.e., the performance more quickly converged under other approaches and ultimately achieved a comparable performance.

The proposed method performed well on extremely imbalanced remote sensing datasets regardless of the data complexity.

## 5. Discussion

To explore the characteristics and advantages of the proposed ISS-XGB, this section discusses the influence of unlabeled data (section A), the utility of the semi-supervised learning strategy (section B), and develops comparisons with methods at the data level in imbalanced learning.

### 5.1. The Influence of Unlabeled Data on ISS-XGB

Unlabeled samples can provide supplementary information about the non-target class, the influence of which is not the same under different imbalanced situations.

When the samples were extremely imbalanced, ISS-XGB had a significant advantage in identifying the minority class. The methods that adopted AIO strategies (RF, XGB, and MLP) were always partial to the majority class, generating biased results. ISS-XGB broke up multi-class problems into several binary-class classification tasks, ensuring that the minority class received sufficient consideration. Thus, the accuracy of ISS-XGB converged quickly to a higher value. Although this strategy was quite similar to that for SVM, the unlabeled samples used in ISS-XGB offered extra distribution information while the negative samples in SVM did not. In particular, when the samples suffered from extreme rarity, SVM failed to fit in the hyperplane and made almost entirely incorrect predictions (Figure 3 and Figure 4b, Table 2 and Table 3). Additionally, the unlabeled data in the training process of ISS-XGB provided the general information of the data to remedy the incomplete description of the target class by rare positive samples. Thus, ISS-XGB provided higher probability prediction for the minority class than the other methods (Figure 3a–e).

**Figure 8:**
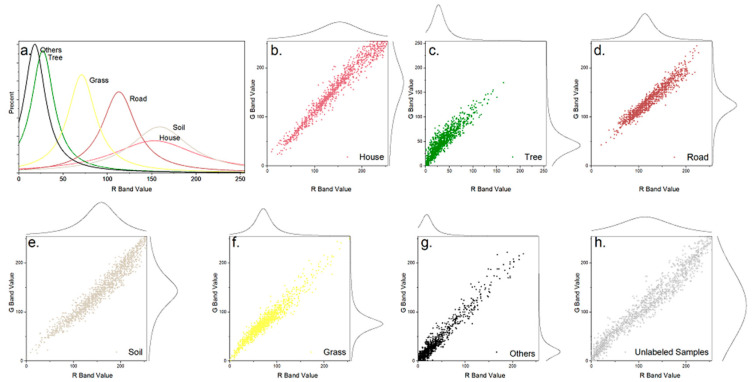
Spectral spaces, consisting of the R-band and G-band, for Area 6 with balanced samples. The x-axis represents the R band, and the y-axis represents the G band. Sub-figure shows the spectral overlap on the R-band in the same coordinate space for all classes. Sub-figure (**a**) shows the percent distribution of R band values for all six classes. Sub-figure (**b**–**g**) shows the feature distribution of each class in this R-G space ((**b**): House; (**c**): Tree; (**d**): Road; (**e**): Soil; (**f**): Grass; (**g**): Others). Sub figure (**h**) shows the distribution of unlabeled samples in the R-G space.

When the data were relatively balanced, the advantages of ISS-XGB were not significant. For further explanation, the 2D spectral spaces of each class in Area 6 were drawn (Figure 8). As the data tended to be balanced, the descriptive ability of the target samples improved (Figure 8b–g). The unlabeled samples were likely less intense or had a negative influence because the unlabeled data represented a mixed class. The features of the data inevitably reflected the target class (Figure 8h), which blurred the distinctions between the unlabeled data and the target samples. Thus, when the positive samples were sufficient, the unlabeled samples interfered with PU heterogeneity and suppressed the class description. Under this situation, the distinction of the positive–positive scheme was better, resulting in a more accurate performance (e.g., XGB and RF in Figure 6 and Figure 7) and higher possibility prediction (Figure 3f–j).

### 5.2. Comparison with PU-BP and PU-SVM

ISS-XGB, PU-BP, and PU-SVM are all semi-supervised methods based on the PU learning framework. The main differences between ISS-XGB and the other two are the strategy patterns for unlabeled data and the positive–unlabeled simulator. For the contrastive analysis, the same training samples from Areas 6 and 8 were employed. Details of PU-BP and PU-SVM can be found in [50,55].

**Figure 9:**
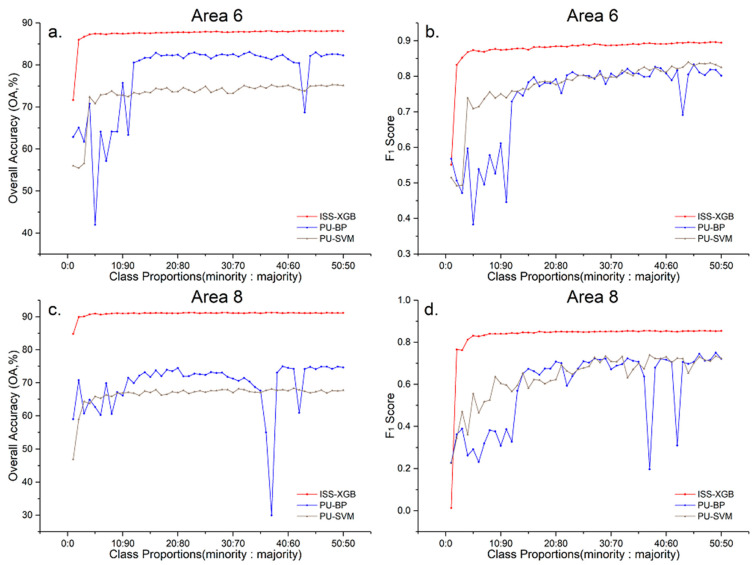
OA and F_1_ score curves for the minority classes in Areas 6 and 8 across different imbalanced samples with ISS-XGB (red), PU-BP (blue), and PU-SVM (grey). Sub-figure (**a**,**b**) are the accuracy curves for Area 6 ((**a**): OA; (**b**): F_1_ score). Sub-figure (**c**,**d**) are the accuracy curves for Area 8 ((**c**): OA; (**d**): F_1_ score).

Figure 9 shows the OA and F_1_ scores for the minority classes of the three methods under different imbalanced situations (i.e., the ratio of the minority and majority from 1:99 to 50:50). ISS-XGB (the red line) had higher and more stable accuracy than PU-BP and PU-SVM. Notably, when the ratio of the minority and the majority was under an extremely imbalanced situation (i.e., from 1:99 to 10:90), the F_1_ score of ISS-XGB for the minority class was an average of 0.14 and 0.10 greater than that of PU-BP and PU-SVM. 

Unlabeled data were used in all three methods, but different usages resulted in significant differences in accuracy. In PU-BP, 5000 unlabeled data were employed, which was more than 5 times the number of positive samples for the minority. In this case, imbalanced learning still existed, while the simulator of PU-BP (i.e., BP) was sensitive to the skewed sample distribution. Thus, the curves of PU-BP (the blue line) strongly fluctuated in their OA and F_1_ scores. In PU-SVM, although the same amount of unlabeled data and positive samples was used, this amount was not sufficient to provide enough information to construct the hyperplane. Therefore, the accuracy under PU-SVM was much lower than that under ISS-XGB (nearly 20% for OA, and approximately 15% for the F_1_ score of the minority class). In ISS-XGB, impartial and multiple positive–unlabeled datasets were constructed. Optimization of the strategy pattern and simulator of ISS-XGB helped the characteristics of the minority be learned fairly and comprehensively.

### 5.3. Comparison with SMOTE Sampling-Based Methods

SMOTE is a popular method in imbalanced learning [25]. This method uses *k* minority class nearest neighbors to generate synthetic samples by operating in feature space [16], which can be implemented directly at the data level without any modifications to the algorithms. 

A comparison was conducted between ISS-XGB and SMOTE-based XGB (SXGB), RF (SRF), MLP (SMLP), and SVM (SSVM). Five neighbors and 100% of the class proportion were set to create new balanced sample sets via SMOTE. Then, the classifiers were trained with generated balanced sets. 

**Figure 10:**
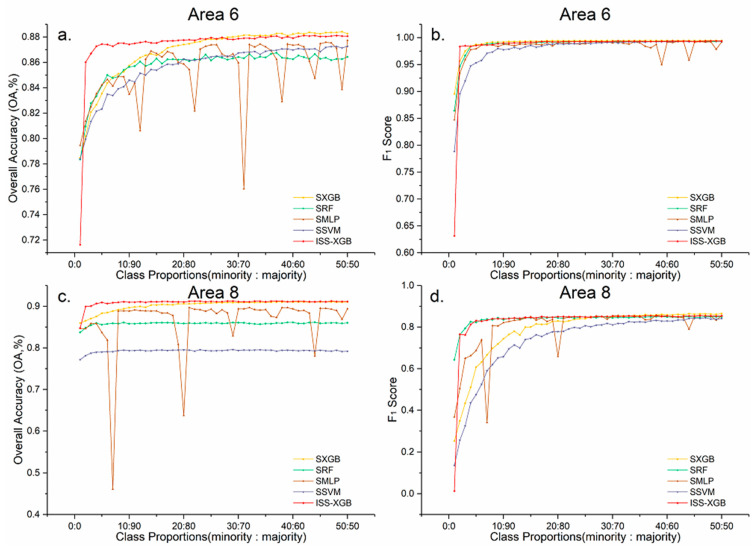
OA and F_1_ score curves for the minority classes in Areas 6 and 8 across different imbalanced samples with ISS-XGB (red), SXGB (yellow), SRF (green), SMLP (brown), and SSVM (purple). Sub-figure (**a**,**b**) are the accuracy curves for Area 6 ((**a**): OA; (**b**): F_1_ score). Sub-figure (**c**,**d**) are the accuracy curves for Area 8 ((**c**): OA; (**d**): F_1_ score).

Areas 6 and 8 were chosen as examples for discussion (Figure 10). In general, ISS-XGB (the red line) provided comparable (even higher) performance to the SMOTE-based methods. As shown in Figure 10c, the highest OA of ISS-XGB (91.25%) is similar to that of SXGB (the yellow line, 91.07%) and 5.23% and 12.06% higher than that of SRF (the green line) and SSVM (the blue line), respectively. Moreover, the OA and F_1_ scores of ISS-XGB were relatively more stable than those of the SMOTE-based methods. For instance, in Area 6, the OA of SMLP (the brown line) fluctuated between 0.76 and 0.87 dramatically, while that of ISS-XGB quickly converged to approximately 0.88 (Figure 10a). Under an extremely imbalanced situation (i.e., class proportions ranging from 1:99 to 10:90), the advantages of ISS-XGB were more obvious. The difference of the F_1_ score between ISS-XGB and SXGB and SSVM was at least 0.10 (Figure 10d).

SMOTE synthesized extra samples for the minority class but was not adopted in ISS-XGB because, in remote sensing datasets, each sample corresponds to a certain object, while SMOTE-synthesized samples may not be related to any objects. The synthesized samples may produce uncertainty in the feature space, which could greatly impact some classifiers (e.g., SMLP). Thus, in ISS-XGB, cost-free unlabeled data were introduced to provide extra information on the overall distribution, rather than the synthesized positive data for the minority class. In addition, since the positive samples of the minority class are extremely rare in many scenarios, multiple unlabeled data sets (10 in this paper) were used for impartial semi-supervised training.

## 6. Conclusions

This study proposed an impartial semi-supervised learning strategy (i.e., ISS-XGB) for land-cover classification with imbalanced remote sensing data. The results generated from the eight study areas demonstrated that ISS-XGB can effectively identify minority classes, especially under extremely imbalanced situations. This method provided high accuracy for the minority class without degrading overall performance and was robust to data complexity. The cost-free unlabeled data utilized in ISS-XGB could provide extra information about the non-target class. Moreover, compared with other semi-supervised methods based on the PU learning framework, optimizing in the strategy patterns and simulations of ISS-XGB can help the characteristics of the minority be learned more fairly and comprehensively. This work provides a new strategy for solving imbalanced classification problems in remote sensing. In the future, combinations of multiple strategies with ISS-XGB and computational efficiency will be further investigated. 

## Figures and Tables

**Table 1:** SHDI (Shannon diversity index) and species of the 8 study areas (boldface in the table indicates the minority class in the corresponding experiment).

Area	SHDI	Species
1	0.83	Farmland with crops, Farmland without crops, Soil
2	0.94	**House**, Tree, Farmland with crops, Farmland without crops, Others
3	1.02	**Tree**, Farmland with crops, Farmland without crops, Soil, Water, Others
4	1.19	Tree, Farmland with crops, Farmland without crops, **Soil**, Grass
5	1.21	**House**, Tree, Farmland with crops, Farmland without crops, Soil, Others
6	1.43	**House**, Tree, Road, Soil, Grass, Others
7	1.67	**House**, Tree, Farmland with crops, Farmland without crops, Soil, Grass, Others
8	2.22	Water, Road, **Tree**, Buildings, Grass, Waterweeds, High-light Objects, Soil, Others (Buildings include three types of building roofs with different colors in pseudo mode)

**Table 2:** Quantity accuracy of approaches with sample sets of different imbalances by MLP (multilayer perceptron), SVM (support vector machine), RF (random forest), XGB (eXtreme gradient boosting), and ISS-XGB (impartial semi-supervised learning strategy based on extreme gradient boosting).

Models	Quantity Accuracy of Models with Sample Sets of Different Imbalances							
	Minority:Majority = 2:98				Minority:Majority = 50:50			
	F_1_	|Z|	QD’ (%)	AD’ (%)	F_1_	|Z|	QD’ (%)	AD’ (%)
MLP	0	31.39 *	100	0	0.86	28.30 *	7.71	26.88
SVM	0	30.05 *	100	0	0.84	25.47 *	2.69	28.67
RF	0	21.04 *	100	0	0.82	12.76 *	0	30.82
XGB	0.01	12.12 *	99.64	0	0.83	1.59	3.23	25.45
ISS-XGB	0.77	-	5.02	37.99	0.85	-	2.69	26.52

* indicates that accuracies differ at a 95 percent level of confidence compared with the performance of ISS-XGB. The QD’/AD’ of the minority class is based on the confusion matrix. QD’/AD’ are the quantity/allocation disagreement number divided by the amount of the minority class in the reference data.

**Table 3:** Average accuracies and standard deviations of Area 6 for 100 trials of 5 approaches.

Models	Average Accuracies and Standard Deviations with Sample Sets of Different Imbalances							
	Minority:Majority = 2:98				Minority:Majority = 50:50			
	OA (Avg./STD)		F_1_ (Avg./STD)		OA (Avg./STD)		F_1_ (Avg./STD)	
MLP	74.65%	/0.0077	0	0	84.87%	/0.0093	0.8343	/0.0198
SVM	74.30%	/0.0075	0	0	84.96%	/0.0093	0.8491	/0.0180
RF	75.62%	/0.0083	0.0424	/0.0588	85.69%	/0.0093	0.8361	/0.0240
XGB	76.81%	/0.0099	0.0895	/0.0864	87.85%	/0.0075	0.8944	/0.0098
ISS-XGB	85.92%	/0.0080	0.8333	/0.0179	87.69%	/0.0077	0.8899	/0.0100

**Table 4:** Confusion matrix of Area 6 for one trial of 5 approaches.

Reference		Confusion Matrix of Classification with Sample Sets of Different Imbalances (Prediction)											
		Minority:Majority = 2:98						Minority:Majority = 50:50					
		House	Tree	Road	Soil	Grass	Others	House	Tree	Road	Soil	Grass	Others
MLP (|Z| = 21.52 *)	House	0	10	451	283	20	28	560	11	147	50	14	10
	Tree	0	798	4	6	80	61	1	807	4	6	73	58
	Road	0	3	907	33	3	2	28	3	887	28	2	0
	Soil	0	4	9	864	72	7	18	5	14	840	73	6
	Grass	0	76	16	58	497	14	18	78	15	45	496	9
	Others	0	70	6	9	9	951	13	79	2	7	8	936
		OA = 75.07% QD = NaN AD = NaN						OA = 84.58% QD = 8.05% AD = 6.52%					
SVM (|Z| = 21.77 *)	House	0	6	551	176	27	32	616	6	91	48	20	11
	Tree	0	791	4	5	89	60	1	792	4	5	89	58
	Road	0	3	910	30	3	2	39	3	880	23	3	0
	Soil	0	5	18	844	85	4	24	6	18	823	82	3
	Grass	0	83	20	46	503	9	20	86	7	39	502	7
	Others	0	75	4	8	9	949	12	82	3	7	6	935
		OA = 74.70% QD = NaN AD = NaN						OA = 84.99% QD = 7.60% AD = 6.80%					
RF (|Z| = 20.98 *)	House	2	6	226	366	164	28	570	6	143	46	17	10
	Tree	0	802	3	6	72	66	1	806	4	6	72	60
	Road	0	3	908	34	2	1	31	2	883	28	4	0
	Soil	0	3	14	861	73	5	22	4	7	845	74	4
	Grass	0	74	7	55	520	5	20	75	6	45	511	4
	Others	0	67	3	11	8	956	13	62	2	7	7	954
		OA = 75.67% QD = 18.44% AD = 4.64%						OA = 85.39% QD = 7.35% AD = 6.59%					
XGB (|Z| = 18.56 *)	House	32	8	259	250	207	36	697	6	23	47	8	11
	Tree	0	813	4	5	62	65	1	812	4	7	61	64
	Road	0	4	917	21	6	0	48	3	876	19	2	0
	Soil	1	5	11	857	78	4	19	5	10	841	77	4
	Grass	0	73	7	51	524	6	18	73	5	50	511	4
	Others	0	49	3	8	12	973	11	50	3	7	8	966
		OA = 76.92% QD = 14.30% AD = 5.75%						OA = 87.89% QD = 7.11% AD = 5.94%					
ISS-XGB (|Z| = 6.80 *)	House	697	8	24	35	16	12	700	6	20	49	7	10
	Tree	1	820	4	6	63	55	1	821	4	6	61	56
	Road	83	3	846	13	2	1	69	3	855	19	2	0
	Soil	140	5	7	734	66	4	17	5	9	849	72	4
	Grass	19	72	4	52	509	5	19	70	4	47	516	5
	Others	12	55	2	8	5	963	12	52	2	8	7	964
		OA = 85.38% QD = 7.26% AD = 8.15%						OA = 87.93% QD = 7.16% AD = 5.99%					

* indicates that the accuracy of one approach differs at a 95 percent level of confidence with different imbalanced samples. QD and AD in Table 4 are the overall quantity disagreement incorporating all 6 classes using the population matrix [67]. Because SVM and MLP fail at predicting the house class with imbalanced samples (2:98), the conversion of the observed sample matrix into an estimated unbiased population matrix is invalid. Thus, the overall QD and AD are invalid.
